# UK Medical Cannabis Registry: A Clinical Outcomes Analysis for Autism Spectrum Disorder

**DOI:** 10.1002/npr2.70146

**Published:** 2026-07-01

**Authors:** Arushika Aggarwal, Simon Erridge, Madhur Varadpande, Evonne Clarke, Katy McLachlan, Ross Coomber, Muhammed Asghar, Urmila Bhoskar, Matthieu Crews, Andrea De Angelis, Muhammad Imran, Fariha Kamal, Laura Korb, Gracia Mwimba, Simmi Sachdeva‐Mohan, Gabriel Shaya, James J. Rucker, Mikael H. Sodergren

**Affiliations:** ^1^ Medical Cannabis Research Group Imperial College London London UK; ^2^ Curaleaf Clinic London UK; ^3^ St. George's Hospital NHS Trust London UK; ^4^ Department of Psychological Medicine Kings College London London UK; ^5^ South London & Maudsley NHS Foundation Trust London UK

**Keywords:** anxiety, autism spectrum disorder, cannabidiol, cannabis, tetrahydrocannabinol

## Abstract

**Introduction:**

Autism spectrum disorder (ASD) is a neurodevelopmental disorder associated with distressed behaviors and psychological challenges. This study aims to evaluate the change in health‐related quality of life (HRQoL), anxiety, and sleep quality in autistic individuals prescribed cannabis‐based medicinal products (CBMPs).

**Method:**

This observational case series analyzed data from the UK Medical Cannabis Registry on autistic adults treated with CBMPs. Demographic and clinical data were collected at baseline, with patient‐reported outcome measures assessed up to 18 months. Primary outcomes included changes in anxiety (GAD‐7), sleep quality (SQS), and HRQoL (EQ‐5D‐5L). Secondary outcomes included the incidence of adverse events. Statistical significance was indicated by *p* < 0.050.

**Results:**

One‐hundred and thirty individuals met the inclusion criteria. GAD‐7 (*p* < 0.001) and SQS (*p* < 0.001) scores improved from baseline to 18 months. EQ‐5D‐5L index values showed improvement from baseline (0.43 ± 0.30) to 18 months (0.51 ± 0.32, *p* < 0.001), and PGIC scores increased from 1 month (5.43 ± 1.49) to 18 months (5.65 ± 1.32, *p* = 0.013). Twenty‐five participants (19.23%) reported a total of 232 (178.46%) adverse events, with most being mild (*n* = 88; 67.69%) or moderate (*n* = 99; 76.15%).

**Conclusion:**

Treatment with CBMPs was associated with improvements in HRQoL, anxiety, and sleep outcomes in autistic patients over an 18‐month period. Given the absence of a control group, these findings represent associations rather than proven treatment effects. Further high‐quality randomized controlled trials are needed to confirm the long‐term efficacy and safety of CBMPs in ASD.

## Abstract 2

A case series studying outcomes up to 18 months in autistic patients from the UK Medical Cannabis Registry.

## Graphical abstract

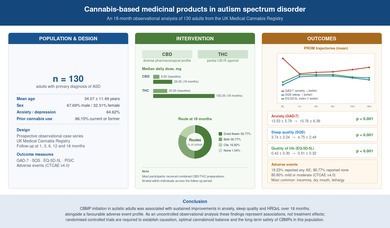

## 1 Introduction

Autism spectrum disorder (ASD) is a neurodevelopmental disorder, marked by ongoing challenges in social communication and interaction, along with the presence of restricted, repeated behaviors, interests or activities [1]. The global prevalence of ASD is estimated to be 0.6%, and is increasing [2]. In addition to the core features of ASD, individuals may display distressed behaviors, such as aggression towards self and others, and experience psychiatric comorbidities, such as anxiety or depression [3, 4]. These behaviors often persist into adulthood, and a study found that 69% of their adolescent and adult patients continued to experience distressed behaviors, including self‐injurious behavior and stereotypy [5]. Individuals with ASD also experience co‐occurring comorbidities including sleep disorders, affecting an estimated 50% to 83%, which can exacerbate distressed behaviors [6]. As a result, ASD is linked to a lower quality of life in both pediatric and adult patients, as well as their caregivers [7, 8].

ASD treatment does not seek to change the core features of the condition, but rather treat associated distressed behaviors or psychiatric conditions [9]. There is a paucity of high‐quality evidence on the efficacy of these therapies in ASD [10, 11]. Conventional pharmaceutical treatments may include selective serotonin reuptake inhibitors and atypical antipsychotics, such as risperidone and aripiprazole, which have shown to reduce some dimensions of symptoms associated with ASD [12]. However, these medications often have poor tolerability [13, 14], and approximately 30%–50% of patients do not respond adequately [15]. Therefore, there is growing interest in exploring alternative and novel treatments for distressed behaviors or psychiatric conditions associated with ASD, including cannabis‐based medicinal products (CBMPs) [16].

The endocannabinoid system (ECS) is a widespread neuromodulatory network that has been linked to the pathophysiology of ASD and is viewed as a potential target for drug development. The principal receptors of the ECS are cannabinoid receptor‐1 (CB1R) and cannabinoid receptor‐2 (CB2R) [17]. CB1Rs are primarily found in the central nervous system, particularly concentrated in the cortex, hippocampus, amygdala, and cerebellum [18]. They are predominantly located on presynaptic terminals of gamma‐aminobutyric acid (GABA)ergic and glutamatergic neurons [19]. CB1R activation inhibits GABA and glutamate release, a process mediated by the endogenous ligand anandamide [20]. Studies have suggested that pediatric patients with ASD have a reduction in endogenous CB1 agonist, anandamide, compared to controls [21, 22, 23]. This may partly explain the sleep disturbance commonly experienced by those with ASD, as anandamide has been shown to promote stage 3 non‐REM sleep [24]. Furthermore, a murine preclinical study showed that selective loss of CB1R alters social behavior and communication, emphasizing the role of the ECS in ASD [25]. CB1R deficiency has also been linked to co‐morbid psychiatric issues, such as depression, which are commonly seen in ASD and can exacerbate altered social behavior [26]. Reduced CB1R activity has also been shown to impair serotonin feedback mechanisms, increasing emotional lability [27].

The primary active compounds in CBMPs are cannabidiol (CBD) and Δ9‐tetrahydrocannabinol (THC) [28]. CBD functions as a negative allosteric modulator of CB1R [29], and can raise anandamide levels through inhibition of its enzymatic degradation [30, 31]. It is also an agonist of 5‐hydroxytryptamine 1A serotonin receptors [32]. THC, meanwhile, is a partial agonist of CB1R [33]. Whilst further evidence is needed, these phytocannabinoids have shown potential clinical effects in various conditions, including epilepsy [34], chronic pain [35], improving anxiety symptoms [36], and enhancing sleep quality [37]. Consequently, they have been recognized as a promising therapeutic option for managing the variety of symptoms linked with ASD [38, 39].

Clinical research has indicated that CBD produces effects on neural connectivity and glutamate and GABA neurotransmitter signaling in brain regions associated with ASD [40, 41]. Furthermore, a randomized controlled trial involving eight participants aged 8 to 16 years with ASD showed that an 8‐week treatment with 20 mg/kg/day of CBD resulted in improvements in Aberrant Behavior Checklist scores [42]. This included improvements in hyperactivity, irritability, and stereotypic behavior, with similar results corroborated by a study conducted by Barchel et al. [43]. Another study conducted by Aran et al. involving 60 children found that a combination of CBD and THC in a 20:1 ratio resulted in significant improvements: 61% of participants experienced reductions in distressed behaviors, 39% showed decreased anxiety, and 47% exhibited progress in communication [44]. Moreover, CBMPs have demonstrated good tolerability with mild side effects [43, 45].

The data for CBMPs for ASD is promising, and a previous UKMCR study showed improvements in overall health‐related quality of life (HRQoL), sleep, and anxiety symptoms following the initiation of treatment with follow up to 6 months [46]. However, there remains a paucity of high‐quality clinical data regarding the efficacy of this treatment option, particularly in adults. Current studies are constrained by short follow‐up periods and small sample sizes, with many failing to use validated measures to evaluate changes in symptoms over time. Whilst there are promising upcoming clinical trials in pediatric patients (NCT04745026 [47], NCT04520685 [48]), there are currently no randomized controlled trials examining the use of CBMPs for ASD in adult patients. Utilizing real‐world data, this study primarily aims to assess the HRQoL outcomes and incidence of adverse events among adult patients registered with the UK Medical Cannabis Registry (UKMCR), who are prescribed CBMPs for ASD up to 18 months.

## 2 Methods

### 2.1 Study Design and Participants

This study examines a case series of patients receiving CBMPs through the UKMCR for a primary diagnosis of ASD. Since its establishment in 2019, the UKMCR has been compiling pseudonymised data for those prescribed CBMPs across the United Kingdom and Crown Dependencies. Privately owned by Curaleaf Clinic, the UKMCR is the most comprehensive registry of its kind [49].

Patient data is collected through both clinical consultations and a custom electronic reporting system. Written informed consent is obtained from participants before enrolment. Ethical approval for this study was granted to the UKMCR by the Central Bristol Research Ethics Committee (reference: 22/SW/0145).

### 2.2 Data Collection

Inclusion criteria required patients to be undergoing CBMP treatment where ASD was the primary indication and a minimum of 18 years old. Individuals treated with CBMPs for other conditions were included only if the symptomatic focus of treatment was assessed to be primarily related to ASD. Participants were excluded if baseline patient‐reported outcome measures (PROMs) were incomplete or if CBMP treatment had commenced within 18 months prior to the UKMCR data extraction on December 13, 2023.

During the initial consultation, patient demographic details, including gender, age, occupation, and body mass index (BMI) were collected. The Charlson Comorbidity Index was utilized to assign each patient a score informed by their age and recorded medical conditions [50]. A higher index score is linked to a greater mortality incidence at both 1 and 10 years [51]. It is a recognized and validated tool, frequently used to assess comorbidities in registries, facilitating comparisons of comorbidity levels between other studies [51].

Data on tobacco use was recorded in pack years, and alcohol consumption was recorded as weekly units. Cannabis status was classified into three categories: ‘never used,’ ‘ex‐user,’ or ‘current user.’ Participants with a history of cannabis consumption were not obligated to provide proof of abstinence from cannabis prior to starting therapy; however, they were counseled to stop all other forms of cannabis. Cannabis gram years, a metric developed to quantify lifetime cannabis use, was calculated for both current and ex‐users. It is calculated by multiplying the daily mean cannabis intake (in grams) by the duration of use (in years) [52]. The use of non‐prescribed cannabis and other illicit drugs, excluding cannabis, was not documented.

Details about the CBMP prescription, including THC and CBD dosages, formulation, strain, and administration route were recorded from each prescription. Depending on the clinical requirement, patients were offered either oral/sublingual (oils) or vapourised (dried flower) forms of the CBMPs. The CBMP dosage was calculated by multiplying the concentration (mg/g or mg/ml) by the amount prescribed for daily use (g/day or ml/day).

### 2.3 Patient‐Reported Outcome Measures

The primary outcomes assess changes in PROM scores from baseline through follow‐up at 1, 3, 6, 12, and 18 months. These PROMs included the Generalized Anxiety Disorder‐7 (GAD‐7), Single‐Item Sleep Quality Scale (SQS), EuroQol‐5 Dimension‐5 Level (EQ‐5D‐5L), and Patient Global Impression of Change (PGIC). The PGIC was only collected at follow‐up assessments, not at baseline.

Baseline PROMs were completed prior to patients receiving their initial CBMP prescription. These PROMs were reassessed at 1, 3, 6, 12, and 18 months. Comparison between follow‐up months and baseline scores facilitated monitoring of CBMP therapy.

The GAD‐7 is a tool used to screen for generalized anxiety disorder and assess its severity. It contains 7 questions that focus on symptoms experienced in the past two weeks [53]. The total score can range from 0 to 21, with scores of ≥ 5, ≥ 10, and ≥ 15 representing mild, moderate, and severe anxiety, respectively [53]. A change of ≥ 4 points is regarded as the minimal clinically significant difference (MCID) [54].

The SQS evaluates an individual's perception of their sleep quality by asking them to rate their sleep over the past week on a scale from 0 (terrible) to 10 (excellent) [55]. From baseline to follow‐up, the MCID is considered a mean score change of 2.6 [55].

The EQ‐5D‐5L is a tool used to assess health‐related quality of life (HRQoL) across five dimensions: anxiety/depression, mobility, pain/discomfort, self‐care, and usual activities [56, 57]. Each dimension is rated from 1 (no problems) to 5 (extreme problems), and the results are combined into a 5‐digit code, which is converted into an index score [58]. A score of 1 indicates optimal health, while values below 0 reflect a state worse than death [59]. The EQ‐5D‐5L provides a standardized measure of HRQoL, with country‐specific index values [59].

The PGIC is used to evaluate a patient's perceived change in health since starting treatment [60]. Patients rate their improvement or deterioration on a 7‐point scale, with 1 indicating no change or worsening of the condition, and 7 indicating significant improvement [61].

Secondary outcomes involved evaluating the incidence and severity of adverse events (AEs).

### 2.4 Adverse Events

Adverse events were captured through patient self‐reports contemporaneously, during completion of PROMs, or by clinicians during regular follow‐up visits, and were categorized according to the Common Terminology Criteria for Adverse Events version 4.03 [62]. The frequency of each adverse event and its severity are calculated as a proportion of the total study population, rather than the total number of adverse events.

### 2.5 Missing Data

To address missing PROM data resulting from patient dropout or incomplete questionnaires, a baseline observation carried forward (BOCF) strategy was implemented. Under this approach, missing data was replaced with the participants' baseline PROM scores, assuming they would revert to their baseline levels if CBMP therapy was discontinued.

### 2.6 Statistical Analysis

Patient demographics, comorbid conditions, drug and alcohol use, prescribed medications, and adverse events were evaluated using descriptive statistics. The distribution of the data was assessed using the Shapiro–Wilk test.

Parametric data is presented as mean ± standard deviation (SD), whereas nonparametric data is presented as median [interquartile range (IQR)], unless stated otherwise. Frequencies are shown as *n* (%).

A repeated measures analysis of variance (ANOVA) was used to evaluate changes in PROM scores. Post hoc pairwise comparisons with Bonferroni correction were performed on significant results. Type I error was less likely as a result of this analysis [63, 64]. Based on the central limit theorem, PROM data were regarded as parametric [65].

A univariate logistic regression analysis was performed to assess the impact of variables on the likelihood of participants experiencing an adverse event at the 18‐month follow‐up. Additionally, a multivariate analysis was conducted to account for the influence of other variables, providing a more comprehensive understanding of the factors associated with adverse events at 18 months. The results of the logistic regression are presented as odds ratios (ORs) with 95% confidence intervals (CIs).

The statistical analyses were performed with the Statistical Package for the Social Sciences (SPSS; v.29.0.0.0), and graphs were created with GraphPad Prism (v. 9.4.1(350)). Statistical significance was indicated by *p* < 0.050.

## 3 Results

**FIGURE 1:**
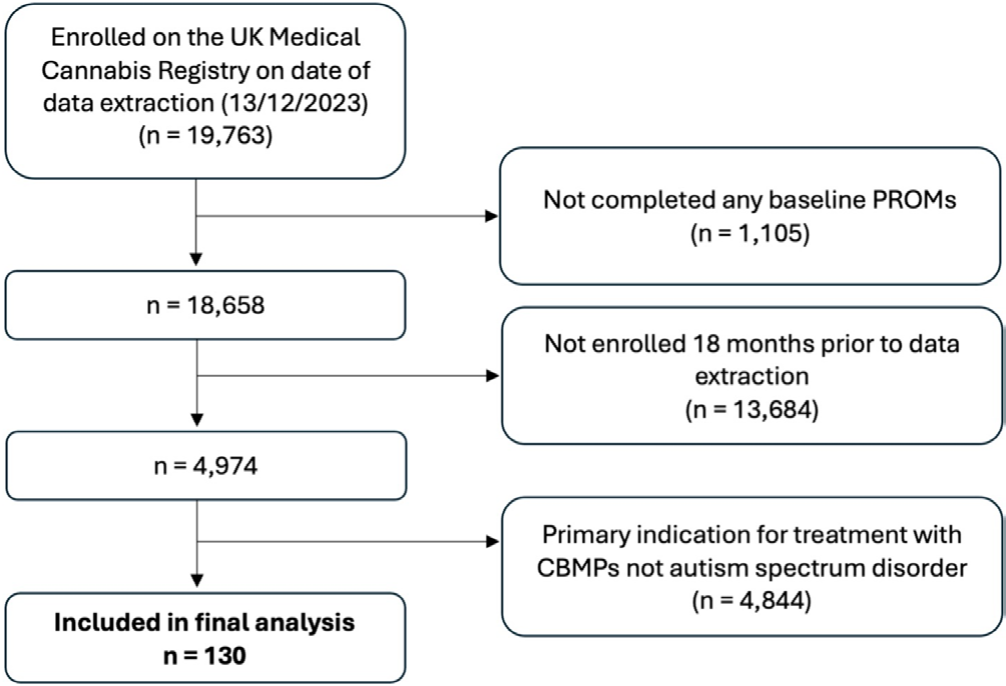
Flowchart of application of study inclusion and exclusion criteria. CBMPs—cannabis‐based medicinal products; PROMs—patient‐reported outcomes measures.

At the time of data extraction on December 13th, 2023, the UKMCR had a total of 19 763 patients. Following the application of inclusion and exclusion criteria, 130 patients were selected for inclusion in the current analysis (Figure 1).

### 3.1 Baseline Demographics

The demographic characteristics of the study participants are summarized in Table 1. A total of 88 (67.69%) of participants were male, while 42 (32.31%) were female. The mean age of participants was 34.07 (± 11.49) years, and the mean BMI was 26.46 (± 7.01) kg/m [2]. Regarding employment status, 57 (43.85%) were employed, while 63 (48.46%) were unemployed.

**TABLE 1:** Baseline demographic characteristics of the study population (*n* = 130).

Baseline demographics	*n* (%)/mean ± SD
Gender	
Male	88 (67.69%)
Female	42 (32.31%)
Age (years)	34.07 ± 11.49
BMI (kg/m^2^)	26.46 ± 7.01
Occupation	
Employed	57 (43.85%)
Clerical support workers	2 (1.54%)
Craft and related trades workers	1 (0.77%)
Elementary occupations	6 (4.62%)
Managers	2 (1.54%)
Other occupations	24 (18.46%)
Professional	9 (6.92%)
Service and sales workers	5 (3.85%)
Skilled agricultural, forestry and fishery workers	1 (0.77%)
Technicians and associate professionals	7 (5.38%)
Unemployed	63 (48.46%)
Unspecified	10 (7.69%)

*Note:* Continuous variables are presented as mean ± standard deviation; categorical variables as *n* (%).

Table 2 presents the medical history of the study population. The median Charlson Comorbidity Index score was 0.00 [0.00–0.00]. The most common comorbidity was ‘anxiety and/or depression,’ reported by 84 participants (64.62%). Thirty‐eight participants (29.23%) had never smoked, 55 (42.31%) were former smokers, and 37 (28.46%) were current smokers. Eighteen participants (13.85%) had never used cannabis, 22 (16.92%) were former users, and 90 (69.23%) were current users at baseline. For current and former cannabis users, the median lifetime consumption was 8.00 [2.00–20.00] gram years.

**TABLE 2:** Medical history of study participants (*n* = 130).

Medical history	*n* (%)/median [IQR]
Charlson Co‐morbidity Index	0.00 [0.00–0.00]
Comorbidities	
Myocardial Infarction	1 (0.77%)
Congestive heart failure	1 (0.77%)
Peripheral vascular disease	0 (0.00%)
Cerebrovascular accident or transient ischemic attack	0 (0.00%)
Dementia	0 (0.00%)
Chronic obstructive pulmonary disease	0 (0.00%)
Connective tissue disease	2 (1.54%)
Peptic ulcer disease	1 (0.77%)
Liver disease	
Mild	2 (1.54%)
Moderate to severe	1 (0.77%)
Diabetes	
None or diet‐controlled	127 (97.69%)
Uncomplicated	3 (2.31%)
Hemiplegia	0 (0.00%)
Moderate to severe chronic kidney disease	1 (0.77%)
Solid tumor	
Metastatic	0 (0.00%)
Localized	6 (4.62%)
Leukemia	0 (0.00%)
Lymphoma	0 (0.00%)
Acquired immunodeficiency syndrome	0 (0.00%)
Hypertension	4 (3.08%)
Anxiety and/or depression	84 (64.62%)
Arthritis	4 (3.08%)
Epilepsy	4 (3.08%)
Venous thromboembolism	0 (0.00%)
Endocrine thyroid dysfunction	5 (3.85%)
Smoking status	
Never smoked	38 (29.23%)
Ex‐smoker	55 (42.31%)
Current smoker	37 (28.46%)
Smoking pack years (current or ex‐smokers)	7.50 [3.00–16.75]
Weekly alcohol consumption (units)	0.00 [0.00–2.00]
Cannabis status	
Never used	18 (13.85%)
Ex‐user	22 (16.92%)
Current user	90 (69.23%)
Cannabis gram years (current or ex‐users)	8.00 [2.00–20.00]

*Note:* This data includes the Charlson Comorbidity Index, reported comorbidities, smoking status, smoking pack years, weekly alcohol consumption, cannabis use status, and cannabis gram years. Data are presented as counts with percentages (%) or as median values with interquartile ranges [IQR].

### 3.2 Cannabis‐Based Medicinal Products

CBMP treatment details, including baseline and maximum titrated doses were recorded for all participants (*n* = 130) (Table 3). Information on administration routes was also documented at baseline and at follow‐ups at 1, 3, 6, 12, and 18 months. The baseline median daily dose of CBD was 8.50 [0.00–11.00] mg/day. The greatest dose increase was at 1 month, to 24.50 [5.00–60.00] mg/day. The highest CBD dose recorded was at 3 months, at 30.00 [5.00–60.00] mg/day, but there was a decrease in dosage after this. By 18 months, the median CBD dose was 26.25 [10.00–60.00] mg/day. The median daily THC dose at baseline was 20.00 [1.00–21.00] mg/day, and this continued to increase at every follow‐up until 18 months, where the highest median dosage was 192.25 [99.45–260.00] mg/day.

**TABLE 3:** Prescribed cannabis‐based medicinal products (CBMPs) (*n* = 130).

CBMP details	Baseline	1‐month	3‐months	6‐months	12‐months	18‐months
	*n* (%)/median [IQR]					
Prescription information						
CBD dosage (mg/day)	8.50 [0.00–11.00]	24.50 [5.00–60.00]	30.00 [5.00–60.00]	20.00 [5.00–60.00]	26.00 [10.00–65.63]	26.25 [10.00–60.00]
THC dosage (mg/day)	20.00 [1.00–21.00]	100.00 [10.95–120.63]	105.00 [11.82–200.00]	151.88 [89.38–207.25]	190.00 [101.88–237.63]	192.25 [99.45–260.00]
Administration routes						
None	0 (0.00%)	2 (1.54%)	4 (3.08%)	2 (1.54%)	4 (3.08%)	2 (1.54%)
No. of patients prescribed oils/	42	36	33	26	20	22
Equivalent	(33.21%)	(27.69%)	(25.38%)	(20.00%)	(15.38%)	(16.92%)
No. of patients prescribed dried flower	61 (46.92%)	57 (43.85%)	59 (45.38%)	63 (48.46%)	67 (51.54%)	66 (50.77%)
No. of patients prescribed both	27 (20.77%)	35 (26.92%)	34 (26.15%)	39 (30.00%)	39 (30.00%)	40 (30.77%)

*Note:* Prescription information is presented as median values with interquartile ranges [IQR], while administration routes are reported as counts with percentages (%).

Abbreviations: CBD—Cannabidiol; THC—Δ^9^‐tetrahydrocannabinol.

The most prescribed administration route at baseline (*n* = 61; 46.92%) and consistently across all follow‐up periods, including 18 months (*n* = 66; 50.77%), was dried flower only.

Curaleaf EMC1 50/< 4 mg/mL CBD/THC (Curaleaf International, United Kingdom) and Curaleaf EMT2 20 mg/mL THC (Curaleaf International, United Kingdom) were the most frequently prescribed CBD‐ and THC‐dominant oils. The most commonly prescribed dried flower was Adven EMT2 20%/< 1% THC/CBD (Curaleaf International, United Kingdom).

### 3.3 Patient‐Reported Outcome Measures

A repeated measures ANOVA was conducted to compare PROM scores across recorded time points (Table 4). Significant changes were demonstrated in PGIC (*p* = 0.013), EQ‐5D‐5L Selfcare (*p* = 0.008), and all other PROMs (*p* < 0.001), except for EQ‐5D‐5L Mobility. Consequently, post hoc pairwise comparisons were performed for PROMs that reported significant changes, with Bonferroni correction to account for multiple comparisons.

**TABLE 4:** Patient‐reported outcome measure scores at baseline and at 1, 3, 6, 12 and 18 months.

PROM	Baseline	1 month	3 months	6 months	12 months	18 months	*p*
GAD‐7	13.53 ± 5.79	8.90 ± 5.58	9.27 ± 6.29	9.46 ± 5.99	10.15 ± 6.42	10.76 ± 6.39	< 0.001***
SQS	3.74 ± 2.24	5.33 ± 2.46	5.48 ± 2.37	5.52 ± 2.56	5.14 ± 2.53	4.75 ± 2.49	< 0.001***
EQ‐5D‐5L Mobility	1.78 ± 1.04	1.69 ± 0.98	1.68 ± 0.93	1.68 ± 0.96	1.76 ± 0.99	1.72 ± 0.97	0.364
EQ‐5D‐5L Selfcare	2.22 ± 1.11	1.98 ± 1.14	2.05 ± 1.10	1.98 ± 1.10	2.07 ± 1.15	2.04 ± 1.20	0.008**
EQ‐5D‐5L							
Usual activities	2.78 ± 1.09	2.16 ± 1.08	2.35 ± 1.05	2.32 ± 1.09	2.38 ± 1.18	2.42 ± 1.14	< 0.001***
EQ‐5D‐5L Pain/							
discomfort	2.44 ± 1.21	2.12 ± 1.07	2.02 ± 0.99	2.06 ± 0.99	2.30 ± 1.14	2.30 ± 1.17	< 0.001***
EQ‐5D‐5L Anxiety/							
depression	3.44 ± 1.22	2.82 ± 1.13	2.75 ± 1.14	2.83 ± 1.14	2.86 ± 1.27	2.98 ± 1.28	< 0.001***
EQ‐5D‐5L							
Index values	0.43 ± 0.30	0.57 ± 0.28	0.58 ± 0.26	0.58 ± 0.27	0.53 ± 0.31	0.51 ± 0.32	< 0.001***
PGIC		5.43 ± 1.49	5.69 ± 1.22	5.54 ± 1.43	5.80 ± 1.31	5.65 ± 1.32	0.013*

*Note:* The included PROMs are GAD‐7 (Generalized Anxiety Disorder‐7), SQS (Sleep Quality Scale), EQ‐5D‐5L (European Quality of Life Five‐Dimension, Five‐Level) dimensions (Mobility, Self‐Care, Usual Activities, Pain/Discomfort, Anxiety/Depression, and Index Values), and PGIC (Patient Global Impression of Change). Values are mean ± standard deviation. *p*‐values are derived from repeated measures analysis of variance.

**FIGURE 2:**
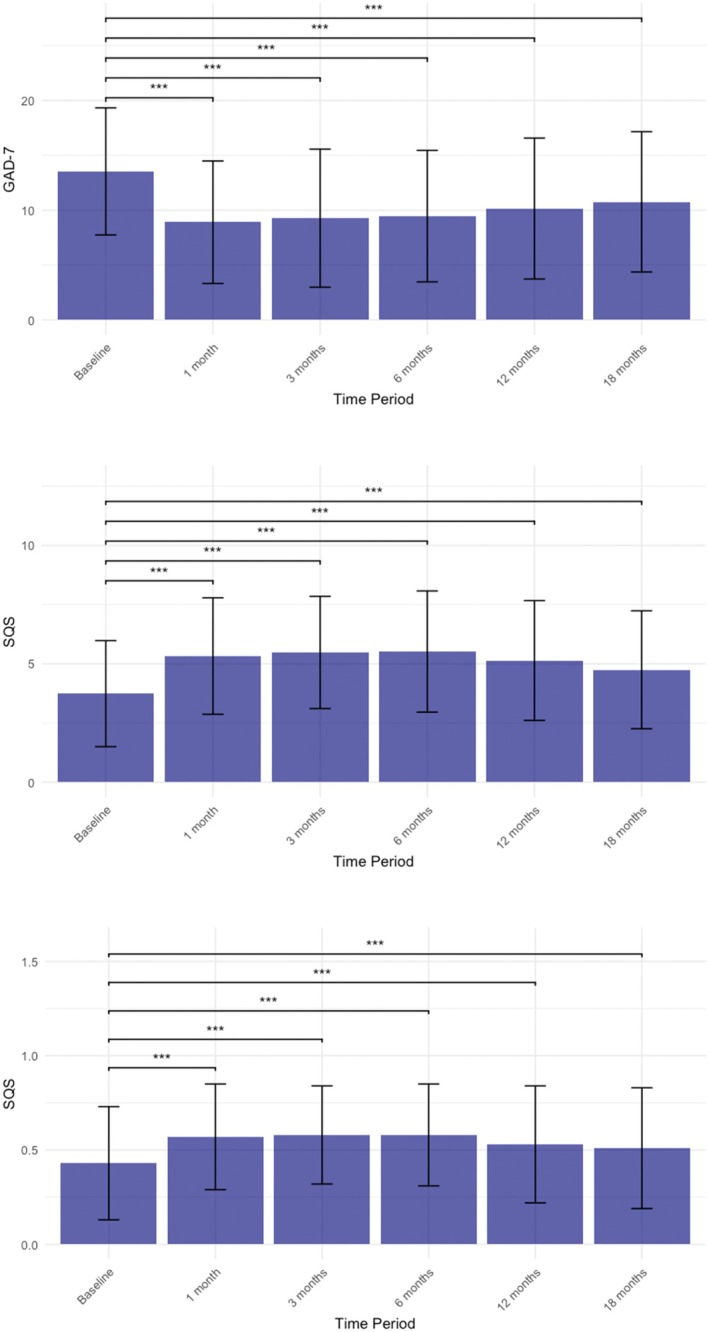
Longitudinal trajectories of patient‐reported outcome measures over 18 months. Mean (± SD) values for the (a) Generalized Anxiety Disorder‐7 (GAD‐7), (b) Single‐Item Sleep Quality Scale (SQS), and (c) EuroQol‐5 Dimension‐5 Level (EQ‐5D‐5L) index value are plotted at baseline, 1, 3, 6, 12, and 18 months. *p < 0.050; **p < 0.010; ***p < 0.001; ns—non‐significant in pairwise comparison between baseline value and follow up time period with Bonferroni correction.

The outcomes of the post hoc pairwise comparisons are displayed in Appendix A, B, C, D, E, F, G. GAD‐7 (Figure 2a), SQS (Figure 2b), EQ‐5D‐5L Usual Activities and EQ‐5D‐5L Index Values (Figure 2c) scales all showed significant improvement from baseline to 1, 3, 6, 12, and 18 month follow‐up periods (*p* < 0.001). EQ‐5D‐5L Anxiety/Depression scores showed greatest improvement at 18 month follow‐up compared to baseline (*p* = 0.007).

### 3.4 Adverse Events

Twenty‐five participants (19.23%) reported 232 (178.46%) adverse events (Table 5). The most common adverse events were insomnia (*n* = 18; 13.85%), dry mouth (*n* = 16; 12.31%) and lethargy (*n* = 16; 12.31%). The majority of reported cases were considered mild (*n* = 88; 67.69%) and moderate (*n* = 99; 76.15%). There were 42 (32.31%) events which were considered severe, and 3 (2.31%) were life‐threatening/disabling.

**TABLE 5:** Incidence and severity of reported adverse events.

Adverse event	Mild	Moderate	Severe	Life‐threatening/Disabling	Total (%)
Abdominal pain	1	1	1	0	3 (2.31%)
Agitation	0	0	1	0	1 (0.77%)
Allergic rhinitis	0	1	0	0	1 (0.77%)
Amnesia	2	3	1	0	6 (4.62%)
Anorexia	1	4	4	0	9 (6.92%)
Anxiety	0	0	0	1	1 (0.77%)
Ataxia	3	1	1	0	5 (3.85%)
Blurred vision	2	2	2	0	6 (4.62%)
Body odor	0	1	0	0	1 (0.77%)
Cognitive disturbance	1	2	2	0	5 (3.85%)
Concentration impairment	8	5	1	0	14 (10.77%)
Confusion	2	0	2	0	4 (3.08%)
Constipation	1	1	0	0	2 (1.54%)
Dehydration	1	0	0	0	1 (0.77%)
Delirium	3	0	1	0	4 (3.08%)
Depression	0	0	1	2	3 (2.31%)
Diarrhea	0	1	0	0	1 (0.77%)
Dizziness	2	2	2	0	6 (4.62%)
Dry mouth	15	1	0	0	16 (12.31%)
Dysgeusia	2	3	0	0	5 (3.85%)
Dyspepsia	5	4	0	0	9 (6.92%)
Fall	0	2	0	0	2 (1.54%)
Fasciculations	0	1	0	0	1 (0.77%)
Fatigue	6	3	4	0	13 (10.00%)
Fever	0	1	0	0	1 (0.77%)
Generalized muscle weakness	1	4	1	0	6 (4.62%)
Headache	1	9	1	0	11 (8.46%)
Insomnia	4	7	7	0	18 (13.85%)
Lethargy	5	11	0	0	16 (12.31%)
Lung infection	0	4	0	0	4 (3.08%)
Nausea	5	2	0	0	7 (5.38%)
Paranoia	0	0	1	0	1 (0.77%)
Pharyngitis	0	4	0	0	4 (3.08%)
Non‐specific rash	2	0	0	0	2 (1.54%)
Seizure	0	1	2	0	3 (2.31%)
Somnolence	0	10	1	0	11 (8.46%)
Spasticity	2	3	1	0	6 (4.62%)
Stereotypies	0	0	1	0	1 (0.77%)
Tics	1	0	0	0	1 (0.77%)
Tremor	5	0	2	0	7 (5.38%)
Varicocele	0	0	1	0	1 (0.77%)
Vertigo	1	2	1	0	4 (3.08%)
Vomiting	2	0	0	0	2 (1.54%)
Weight loss	4	3	0	0	7 (5.38%)
Total (%)	88 (67.69%)	99 (76.15%)	42 (32.31%)	3 (2.31%)	232 (178.46%)

*Note:* Counts represent the number of events at each severity grade (CTCAE v4.0). The “Total (%)” column is the number and percentage of adverse events represented as a proportion of the total cohort (*n* = 130) experiencing each adverse event at least once. Twenty‐five participants (19.23%) reported 232 (178.46%) adverse events.

### 3.5 Univariate and Multivariate Analysis

A univariate logistic regression analysis was performed to assess the association between variables and a participant's likelihood of experiencing an adverse event at 18‐month follow up (Appendix H). These variables included gender, age, BMI, Charlson co‐morbidity index score, cannabis status, CBMP route of administration, and total THC and CBD dosages. No specific variable was associated with an increased odds of experiencing an adverse event (*p* > 0.050).

All variables were taken forward into a multivariate analysis (Appendix I). Again, this showed no relationship between the examined variables and an increased likelihood of experiencing an adverse event (*p* > 0.050).

## 4 Discussion

This case series explored the outcomes of autistic adults treated with CBMPs. The findings indicate that CBMPs were associated with improvements in anxiety, sleep quality, and health‐related quality of life. While a proportion (19.23%) of the cohort reported adverse events, the majority were mild or moderate. As an uncontrolled observational analysis, the study cannot establish causation; reported changes should be interpreted as temporal associations and not as evidence of a treatment effect.

The present findings align with previous research demonstrating the potential benefits of CBMPs in ASD. Systematic reviews by Silva et al. and Poli et al. have reported improvements in quality of life and reductions in ASD‐related symptoms, including sleep disturbances, hyperactivity, and distressed behaviors [38, 66]. Consistent with these reviews, this study reported improvements in sleep quality, anxiety, and HRQoL. Furthermore, the results corroborate findings from a prior study by our group from the UKMCR, which showed positive outcomes in a cohort of 74 ASD patients treated with CBMPs for up to six months [46]. Notably, this current study extends our previous work by reporting outcomes over an 18‐month follow‐up period, providing longer‐term insights. Adding to the growing body of evidence, a recent retrospective analysis of 20 autistic individuals prescribed full‐spectrum CBMP extracts also reported improvements in HRQoL and core and comorbid symptoms. This reinforces the potential of CBMPs that are associated or can co‐occur with ASD [67].

Anxiety is prevalent in ASD, with studies indicating that nearly 40% of young individuals meet the criteria for at least one DSM‐IV classified anxiety disorder [68]. Consistent with this, the most prevalent comorbidity observed in the present study was also anxiety and/or depression. This heightened anxiety is thought to arise from a combination of sensory sensitivities and socio‐communication impairments, resulting in social anxiety [69]. The present study demonstrated a reduction in generalized anxiety symptoms among ASD patients, particularly from baseline (13.53 ± 5.79) to 1 month (8.90 ± 5.58). This aligns with evidence that cannabinoids, through their interaction with the endocannabinoid system, may have anxiolytic effects [70] and a study by *Sethi* et al. proposed that this mechanism may be mediated through the benzodiazepine binding site on central GABA receptors [71]. However, the magnitude of difference in GAD‐7 scores compared to baseline (13.53 ± 5.79) decreased from three (9.27 ± 6.29) to 18 months (10.76 ± 6.39). One potential explanation for this attenuated effect is the development of tolerance to the medication, specifically to the anxiolytic effects of THC, a major component of the CBMPs prescribed in the present study [72]. At 18 months, the median THC dose was 192.25 mg/day, highlighting the potential for tolerance. This suggests that sustained or complementary therapeutic strategies may be necessary to maintain long‐term benefits. Furthermore, the use of the BOCF method to handle missing data might have biased the results towards a null finding. Whilst this provides the most conservative measure of effect of CBMPs, this will have a greater effect on later outcomes which are the most subject to participant attrition. These findings are also consistent with a previous UKMCR study focussing on CBMP therapy for anxiety [73]. This underscores the complex relationship between ASD and anxiety and suggests a cautious but promising role for CBMPs in managing anxiety symptoms in this population.

Sleep disturbances are a significant concern for autistic individuals, often exacerbating other symptoms and diminishing overall quality of life. It is estimated that 60% of autistic adults experience poor sleep quality, particularly insomnia, that impacts their quality of life [74]. In the current study, CBMP initiation was associated with improvements in sleep quality, as evidenced by an increase in SQS scores from baseline (3.74 ± 2.24) to 1 month (5.33 ± 2.46), with sustained improvements observed at three and 6 months. These findings are consistent with previous research that has shown positive effects associated with CBMPs on sleep in autistic individuals [75, 76, 77]. This aligns with the understanding that the endocannabinoid system plays a crucial role in regulating the sleep–wake cycle [78]. However, similar to anxiety, the study observed a decline in these improvements at 12 and 18 months. Furthermore, insomnia was the most commonly reported adverse event, a finding corroborated by Aran et al. [44]. This suggests that while CBMPs can positively impact sleep for some autistic individuals, there may be variability in response between individuals.

The median daily THC dose increased nearly tenfold across the follow‐up period, from 20.00 [1.00–21.00] mg/day at baseline to 192.25 [99.45–260.00] mg/day at 18 months. Several factors are likely to have contributed. First, the cohort was predominantly prescribed inhaled dried flower (46.92% at baseline; 50.77% at 18 months), which has substantially lower systemic bioavailability than ingested oils, such that absolute milligram doses are not directly comparable with oral preparations. Second, doses were titrated by specialist clinicians under multidisciplinary review and in line with UK guidance, with the aim of identifying the lowest effective dose for individual patients. Third, the parallel attenuation of GAD‐7 and SQS scores between three and 18 months is consistent with the development of pharmacological tolerance to the anxiolytic and sedative effects of THC, which has been described in both preclinical and clinical settings. The implication for clinical practice is that prescribers should anticipate dose escalation when initiating dried‐flower CBMPs and counsel patients regarding the risks associated with high chronic THC exposure, including dependence, cognitive effects, and cardiometabolic outcomes. Future prospective studies should pre‐specify maximum dose ceilings, incorporate validated measures of tolerance and dependence, and report bioavailability‐adjusted exposure metrics to facilitate cross‐study comparison.

There was a total adverse events incidence of 178.46%, reported by 19.23% of participants. This finding aligns with a previous UKMCR study evaluating the use of CBMPs for ASD [46]. This reported incidence is higher than in other observational series from other settings [79, 80]. This discrepancy is likely attributable to the rigorous methodology employed by the UKMCR, where patients are systematically encouraged to report adverse events at regular intervals (1, 3, 6, 12, and 18 months) and during clinical visits, ensuring comprehensive capture of adverse events. The most frequently reported adverse event was insomnia (*n* = 18; 13.85%). However, relying on self‐reports may have posed challenges, as autistic individuals often experience chronic sleep difficulties, making it difficult to distinguish between treatment‐related effects and underlying symptoms. Similarly, the life‐threatening or disabling adverse events reported, including anxiety and depression, are prevalent comorbidities in ASD, further complicating the differentiation between treatment‐related side effects and symptoms of the condition itself. In general, short‐term CBMP use has demonstrated a good safety profile in other studies [81]. In the present study, 80.77% of patients did not report any adverse events, and the majority of adverse events were mild‐to‐moderate, further supporting this finding. However, the risks associated with long‐term CBMP use remain poorly understood, highlighting the need for higher‐quality trials to assess the safety of prolonged exposure to CBMPs.

This study has several limitations. Primarily, this is an observational case series, which prevents the establishment of any cause‐and‐effect relationships. The absence of a control or placebo group further hinders the ability to definitively attribute observed changes to CBMP treatment, thus reducing the study's internal validity. The specific CBMPs prescribed throughout the study varied and were prescribed according to clinical judgment. This meant, even within the cohort, a range of different products and doses of each major cannabinoid were used. Whilst this provides additional external validity, it hinders the ability to draw direct insights into which doses of THC and CBD are appropriate for autistic patients. Furthermore, the present study relied on general health‐related measures rather than autism‐specific assessments. Incorporating tools like the Autism Spectrum Quality of Life form, or the World Health Organization Quality of Life‐BREF with autism‐specific items would have provided a more nuanced understanding of the impact of CBMPs on this population [82, 83]. The cohort was recruited from a single private specialist clinic, which is likely to over‐represent individuals with the financial means and health literacy to access self‐funded therapy and to under‐represent those from minoritised or socioeconomically deprived groups; this restricts the external validity of the findings to comparable healthcare contexts. However, it is noteworthy that a significant proportion of participants in the study were unemployed (48.46%), possibly due to the challenges posed by ASD. This suggests that unemployment did not necessarily preclude access to treatment. Demographically, the majority of participants were male (67.69%), which aligns with the higher prevalence of ASD in males [84, 85]. A further important consideration is pre‐treatment cannabis exposure. Most (86.15%) of the cohort were current or former users of non‐prescribed cannabis at enrolment. Prior cannabis exposure may bias the cohort towards individuals who have previously experienced symptomatic benefit from cannabis and who therefore self‐select for treatment, potentially inflating apparent improvement [86]. Conversely, chronic prior use may attenuate response through pharmacological tolerance to THC and through carry‐over effects on baseline severity. As a result, the findings of this study may have limited generalisability to the broader population and an adequately powered comparison between cannabis naïve individuals and previous cannabis consumers is required before any clinical recommendations can be made. While this study has a follow‐up period of 18 months among those exploring CBMPs for ASD in adults, additional research with extended follow‐up periods is needed to fully assess the long‐term effects of CBMP treatment, including safety, dosing, and the potential development of tolerance.

## 5 Conclusion

This observational study suggests that CBMP initiation in autistic adults is associated with improvements in HRQoL, anxiety, and sleep quality over 18 months. There was a favorable safety profile, with 80.77% of patients not reporting any adverse events. As the study lacked a control group, randomization, and blinding, the findings should be interpreted as associations only. To strengthen these findings and address the existing knowledge gaps, larger observational studies and RCTs are essential. Nevertheless, this study provides valuable insights that can inform future RCTs and contribute to the development of evidence‐based clinical guidelines for CBMP use in ASD.

## Funding

The authors have nothing to report.

## Ethics Statement

Formal ethical approval for the UK Medical Cannabis Registry has been provided by the Health Research Authority (South West—Central Bristol Research Ethics Committee reference 22/SW/0145).

## Consent

All patients provided informed consent prior to enrolment in the UK Medical Cannabis Registry. All study participants gave formal, informed, and written consent, preceding their consecutive enrollment into the database.

## Conflicts of Interest

The authors declare no conflicts of interest.

## Data Availability

The data are not publicly available due to privacy or ethical restrictions. The number of patients prescribed cannabis‐based medicinal products in the UK is comparatively small, and the combination of baseline demographic characteristics, the specific products and doses prescribed, and the longitudinal outcome data creates a realistic risk of inadvertent re‐identification of individuals. The patients in this study provided informed consent to participate in the research and for their data to be used for the stated analyses. They did not consent to their individual‐level data being made publicly or widely available. De‐identified individual‐level data may be shared with researchers on reasonable request, subject to the requesting party holding appropriate ethical approval and a data sharing agreement, and being able to host the data within a secure environment with the requisite IT protections.

## Appendix A

Post hoc comparison of generalized anxiety disorder‐7 (GAD‐7) scores for each follow‐up period, after significance was determined with the repeated measures analysis of variance (ANOVA). The values represent the mean difference ± standard error. **p* < 0.050, ***p* < 0.010, ****p* < 0.001.

**Table:**

GAD‐7	Baseline	1 month	3 months	6 months	12 months	18 months
Baseline		4.63 ± 0.49; *p* < 0.001***	4.26 ± 0.52; *p* < 0.001***	4.07 ± 0.55; *p* < 0.001***	3.38 ± 0.50; *p* < 0.001***	2.77 ± 0.48; *p* < 0.001***
1 month	−4.63 ± 0.49; *p* < 0.001***		−0.37 ± 0.44; *p* = 1.000	−0.56 ± 0.48; *p* = 1.000	−1.25 ± 0.53; *p* = 0.282	−1.86 ± 0.56; *p* = 0.018*
3 months	−4.26 ± 0.52; *p* < 0.001***	0.37 ± 0.44; *p* = 1.000		−0.19 ± 0.36; *p* = 1.000	−0.89 ± 0.42; *p* = 0.525	−1.49 ± 0.47; *p* = 0.031*
6 months	−4.07 ± 0.55; *p* < 0.001***	0.56 ± 0.48; *p* = 1.000	0.19 ± 0.36; *p* = 1.000		−0.69 ± 0.46; *p* = 1.000	−1.30 ± 0.52; *p* = 0.206
12 months	−3.38 ± 0.50; *p* < 0.001***	1.25 ± 0.53; *p* = 0.282	0.89 ± 0.42; *p* = 0.525	0.69 ± 0.46; *p* = 1.000		−0.61 ± 0.30; *p* = 0.639
18 months	−2.77 ± 0.48; *p* < 0.001***	1.86 ± 0.56; *p* = 0.018*	1.49 ± 0.47; *p* = 0.031*	1.30 ± 0.52; *p* = 0.206	0.61 ± 0.30; *p* = 0.639	

## Appendix B

Post hoc comparison of sleep quality scale (SQS) scores for each follow‐up period, after significance was determined with the repeated measures analysis of variance (ANOVA). The values represent the mean difference ± standard error. **p* < 0.050, ***p* < 0.010, ****p* < 0.001.

**Table:**

SQS	Baseline	1 month	3 months	6 months	12 months	18 months
Baseline		−1.59 ± 0.22; *p* < 0.001***	−1.75 ± 0.22; *p* < 0.001***	−1.79 ± 0.24; *p* < 0.001***	−1.40 ± 0.21; *p* < 0.001***	−1.02 ± 0.20; *p* < 0.001***
1 month	1.59 ± 0.22; *p* < 0.001***		−0.15 ± 0.18; *p* = 1.000	−0.19 ± 0.22; *p* = 1.000	0.19 ± 0.25; *p* = 1.000	0.58 ± 0.25; *p* = 0.354
3 months	1.75 ± 0.22; *p* < 0.001***	0.15 ± 0.18; *p* = 1.000		−0.04 ± 0.17; *p* = 1.000	0.35 ± 0.24; *p* = 1.000	0.73 ± 0.22; *p* = 0.020*
6 months	1.79 ± 0.24; *p* < 0.001***	0.19 ± 0.22; *p* = 1.000	0.04 ± 0.17; *p* = 1.000		0.39 ± 0.22; *p* = 1.000	0.77 ± 0.22; *p* = 0.009**
12 months	1.40 ± 0.21; *p* < 0.001***	−0.19 ± 0.25; *p* = 1.000	−0.35 ± 0.24; *p* = 1.000	−0.39 ± 0.22; *p* = 1.000		0.39 ± 0.17; *p* = 0.350
18 months	1.02 ± 0.20; *p* < 0.001***	−0.58 ± 0.25; *p* = 0.354	−0.73 ± 0.22; *p* = 0.020*	−0.77 ± 0.22; *p* = 0.009**	−0.39 ± 0.17; *p* = 0.350	

## Appendix C

Post hoc comparison of European quality‐of‐life‐5 dimension‐5 level (EQ‐5D‐5L) self care scores for each follow‐up period, after significance was determined with the repeated measures analysis of variance (ANOVA). The values represent the mean difference ± standard error. **p* < 0.050, ***p* < 0.010, ****p* < 0.001.

**Table:**

EQ‐5D‐5L Self care	Baseline	1 month	3 months	6 months	12 months	18 months
Baseline		0.23 ± 0.06; *p* = 0.007**	0.16 ± 0.06; *p* = 0.164	0.24 ± 0.06; *p* = 0.001**	0.15 ± 0.06; *p* = 0.353	0.18 ± 0.06; *p* = 0.051*
1 month	−0.23 ± 0.06; *p* = 0.007**		−0.07 ± 0.07; *p* = 1.000	0.01 ± 0.07; *p* = 1.000	−0.09 ± 0.07; *p* = 1.000	−0.05 ± 0.08; *p* = 1.000
3 months	−0.16 ± 0.06; *p* = 0.164	0.07 ± 0.07; *p* = 1.000		0.08 ± 0.06; *p* = 1.000	−0.02 ± 0.07; *p* = 1.000	0.02 ± 0.07; *p* = 1.000
6 months	−0.24 ± 0.06; *p* = 0.001**	−0.01 ± 0.07; *p* = 1.000	−0.08 ± 0.06; *p* = 1.000		−0.09 ± 0.07; *p* = 1.000	−0.06 ± 0.07; *p* = 1.000
12 months	−0.15 ± 0.06; *p* = 0.353	0.09 ± 0.07; *p* = 1.000	0.02 ± 0.07; *p* = 1.000	0.09 ± 0.07; *p* = 1.000		0.03 ± 0.05; *p* = 1.000
18 months	−0.18 ± 0.06; *p* = 0.051*	0.05 ± 0.08; *p* = 1.000	−0.02 ± 0.07; *p* = 1.000	0.06 ± 0.07; *p* = 1.000	−0.03 ± 0.05; *p* = 1.000	

## Appendix D

Post hoc comparison of European quality‐of‐life‐5 dimension‐5 level (EQ‐5D‐5L) usual activities scores for each follow‐up period, after significance was determined with the repeated measures analysis of variance (ANOVA). The values represent the mean difference ± standard error. **p* < 0.050, ***p* < 0.010, ****p* < 0.001.

**Table:**

EQ‐5D‐5L Usual activities	Baseline	1 month	3 months	6 months	12 months	18 months
Baseline		0.62 ± 0.10; *p* < 0.001***	0.43 ± 0.09; *p* < 0.001***	0.45 ± 0.08; *p* < 0.001***	0.39 ± 0.08; *p* < 0.001***	0.35 ± 0.08; *p* < 0.001***
1 month	−0.62 ± 0.10; *p* < 0.001***		−0.19 ± 0.09; *p* = 0.565	−0.16 ± 0.09; *p* = 0.932	−0.22 ± 0.10; *p* = 0.351	−0.26 ± 0.10; *p* = 0.189
3 months	−0.43 ± 0.09; *p* < 0.001***	0.19 ± 0.09; *p* = 0.565		0.02 ± 0.07; *p* = 1.000	−0.04 ± 0.07; *p* = 1.000	−0.08 ± 0.09; *p* = 1.000
6 months	−0.45 ± 0.08; *p* < 0.001***	0.16 ± 0.09; *p* = 0.932	−0.02 ± 0.07; *p* = 1.000		−0.06 ± 0.08; *p* = 1.000	−0.10 ± 0.08; *p* = 1.000
12 months	−0.39 ± 0.08; *p* < 0.001***	0.22 ± 0.10; *p* = 0.351	0.04 ± 0.07; *p* = 1.000	0.06 ± 0.08; *p* = 1.000		−0.04 ± 0.06; *p* = 1.000
18 months	−0.35 ± 0.08; *p* < 0.001***	0.26 ± 0.10; *p* = 0.189	0.08 ± 0.09; *p* = 1.000	0.10 ± 0.08; *p* = 1.000	0.04 ± 0.06; *p* = 1.000	

## Appendix E

Post hoc comparison of European quality‐of‐life‐5 dimension‐5 level (EQ‐5D‐5L) pain/discomfort scores for each follow‐up period, after significance was determined with the repeated measures analysis of variance (ANOVA). The values represent the mean difference ± standard error. **p* < 0.050, ***p* < 0.010, ****p* < 0.001.

**Table:**

EQ‐5D‐5L Pain/discomfort	Baseline	1 month	3 months	6 months	12 months	18 months
Baseline		0.32 ± 0.09; *p* = 0.005**	0.42 ± 0.08; *p* < 0.001***	0.38 ± 0.09; *p* < 0.001***	0.14 ± 0.08; *p* = 1.000	0.14 ± 0.08; *p* = 1.000
1 month	−0.32 ± 0.09; *p* = 0.005**		0.09 ± 0.07; *p* = 1.000	0.05 ± 0.09; *p* = 1.000	−0.19 ± 0.09; *p* = 0.543	−0.19 ± 0.10; *p* = 0.815
3 months	−0.42 ± 0.08; *p* < 0.001***	−0.09 ± 0.07; *p* = 1.000		−0.04 ± 0.07; *p* = 1.000	−0.28 ± 0.08; *p* = 0.009**	−0.28 ± 0.09; *p* = 0.020*
6 months	−0.38 ± 0.09; *p* < 0.001***	−0.05 ± 0.09; *p* = 1.000	0.04 ± 0.07; *p* = 1.000		−0.24 ± 0.07; *p* = 0.019*	−0.24 ± 0.08; *p* = 0.048*
12 months	−0.14 ± 0.08; *p* = 1.000	0.19 ± 0.09; *p* = 0.543	0.28 ± 0.08; *p* = 0.009**	0.24 ± 0.07; *p* = 0.019		0.00 ± 0.06; *p* = 1.000
18 months	−0.14 ± 0.08; *p* = 1.000	0.19 ± 0.10; *p* = 0.815	0.28 ± 0.09; *p* = 0.020*	0.24 ± 0.08; *p* = 0.048*	0.00 ± 0.06; *p* = 1.000	

## Appendix F

Post hoc comparison of European quality‐of‐life‐5 dimension‐5 level (EQ‐5D‐5L) anxiety/depression scores for each follow‐up period, after significance was determined with the repeated measures analysis of variance (ANOVA). The values represent the mean difference ± standard error. **p* < 0.050, ***p* < 0.010, ****p* < 0.001.

**Table:**

EQ‐5D‐5L Anxiety/depression	Baseline	1 month	3 months	6 months	12 months	18 months
Baseline		0.62 ± 0.10; *p* < 0.001***	0.69 ± 0.10; *p* < 0.001***	0.61 ± 0.10; *p* < 0.001***	0.58 ± 0.09; *p* < 0.001***	0.45 ± 0.09; *p* = 0.007**
1 month	−0.62 ± 0.10; *p* < 0.001***		0.06 ± 0.09; *p* = 1.000	−0.02 ± 0.10; *p* = 1.000	−0.05 ± 0.10; *p* = 1.000	−0.17 ± 0.11; *p* = 1.000
3 months	−0.69 ± 0.10; *p* < 0.001***	−0.06 ± 0.09; *p* = 1.000		−0.08 ± 0.07; *p* = 1.000	−0.11 ± 0.09; *p* = 1.000	−0.23 ± 0.10; *p* = 0.422
6 months	−0.61 ± 0.10; *p* < 0.001***	0.02 ± 0.10; *p* = 1.000	0.08 ± 0.07; *p* = 1.000		−0.03 ± 0.10; *p* = 1.000	−0.15 ± 0.11; *p* = 1.000
12 months	−0.58 ± 0.09; *p* < 0.001***	0.05 ± 0.10; *p* = 1.000	0.11 ± 0.09; *p* = 1.000	0.03 ± 0.10; *p* = 1.000		−0.12 ± 0.07; *p* = 1.000
18 months	−0.45 ± 0.09; *p* < 0.001***	0.17 ± 0.11; *p* = 1.000	0.23 ± 0.10; *p* = 0.422	0.15 ± 0.11; *p* = 1.000	0.12 ± 0.07; *p* = 1.000	

## Appendix G

Post hoc comparison of European quality‐of‐life‐5 dimension‐5 level (EQ‐5D‐5L) index value scores for each follow‐up period, after significance was determined with the repeated measures analysis of variance (ANOVA). The values represent the mean difference ± standard error. **p* < 0.050, ***p* < 0.010, ****p* < 0.001.

**Table:**

EQ‐5D‐5L Index values	Baseline	1 month	3 months	6 months	12 months	18 months
Baseline		−0.14 ± 0.02; *p* < 0.001***	−0.16 ± 0.02; *p* < 0.001***	−0.15 ± 0.02; *p* < 0.001***	−0.10 ± 0.02; *p* < 0.001***	−0.09 ± 0.02; *p* < 0.001***
1 month	0.14 ± 0.02; *p* < 0.001***		−0.01 ± 0.02; *p* = 1.000	−0.01 ± 0.02; *p* = 1.000	0.04 ± 0.02; *p* = 1.000	0.06 ± 0.03; *p* = 0.447
3 months	0.16 ± 0.02; *p* < 0.001***	0.01 ± 0.02; *p* = 1.000		0.01 ± 0.01; *p* = 1.000	0.05 ± 0.02; *p* = 0.113	0.07 ± 0.02; *p* = 0.041*
6 months	0.15 ± 0.02; *p* < 0.001***	0.01 ± 0.02; *p* = 1.000	−0.01 ± 0.02; *p* = 1.000		0.05 ± 0.02; *p* = 0.310	0.06 ± 0.02; *p* = 0.076*
12 months	0.10 ± 0.02; *p* < 0.001***	−0.04 ± 0.02; *p* = 1.000	−0.05 ± 0.02; *p* = 0.113	−0.05 ± 0.02; *p* = 0.310		0.02 ± 0.02; *p* = 1.000
18 months	0.09 ± 0.02; *p* < 0.001***	−0.06 ± 0.03; *p* = 0.447	−0.07 ± 0.02; *p* = 0.041*	−0.06 ± 0.02; *p* = 0.076*	−0.02 ± 0.02; *p* = 1.000	

## Appendix H

Table illustrating the odds ratio (95% confidence interval (CI)), representing the impact of variables on participants' likelihood of experiencing an adverse event at 18‐month follow up. A univariate logistic regression model was utilized to conduct statistical analysis. *n* = 130, except for body mass index where *n* = 126, and CBMP route of administration where *n* = 128. CBD – cannabidiol; CBMP – cannabis‐based medicinal product; THC—Δ^9^‐tetrahydrocannabinol.

**Table:**

Variable	*n*	Odds ratio (95% CI)	*p*
Gender			
Female	42	—	
Male	88	0.815 (0.326–2.034)	0.661
Age (years)			
< 30	45	—	
30–39	48	1.333 (0.500–3.553)	0.565
≥ 40	37	0.485 (0.136–1.725)	0.264
Body mass index (kg/m^2^)			
< 18.5	13	—	
18.5–24.9	49	1.073 (0.199–5.795)	0.935
25–29.9	32	2.152 (0.396–11.690)	0.375
30–34.9	16	1.269 (0.179–9.021)	0.812
≥ 35	16	0.786 (0.095–6.501)	0.823
Charlson co‐morbidity index			
None (0)	103	—	
Mild/moderate (1–4)	12	2.361 (0.641–8.694)	0.196
Severe (≥ 5)	15	1.181 (0.302–4.616)	0.811
Cannabis status			
Never used	18	—	
Current user	90	0.368 (0.119–1.145)	0.084
Ex‐user	22	0.588 (0.145–2.381)	0.457
CBMP route of administration			
Oil/sublingual formulations only	22	—	
Vapourised flower only	66	0.915 (0.287–2.916)	0.881
Both	40	0.600 (0.160–2.250)	0.449
Total CBD dosage			
≤ median dose (26.25 mg/day)	65	—	
> median dose (26.25 mg/day)	65	1.650 (0.680–4.006)	0.269
Total THC dosage			
≤ median dose (192.25 mg/day)	65	—	
> median dose (192.25 mg/day)	65	0.906 (0.378–2.168)	0.824

## Appendix I

Table illustrating the odds ratio (95% confidence interval (CI)), representing the impact of variables on participants' likelihood of experiencing an adverse event at 18‐month follow up. A multivariate logistic regression model was utilized to conduct statistical analysis. *n* = 124. CBD—cannabidiol; CBMP—cannabis‐based medicinal product; THC—Δ^9^‐tetrahydrocannabinol.

**Table:**

Variable	*n*	Odds ratio (95% CI)	*p*
Gender			
Female	41	—	
Male	83	0.836 (0.297–2.351)	0.734
Age (years)			
< 30	41	—	
30–39	48	1.758 (0.446–6.925)	0.420
≥ 40	35	0.191 (0.017–2.131)	0.179
Body mass index (kg/m^2^)			
< 18.5	13	—	
18.5–24.9	49	1.029 (0.160–6.631)	0.976
25–29.9	30	2.708 (0.409–17.925)	0.301
30–34.9	16	1.144 (0.138–9.521)	0.901
≥ 35	16	0.633 (0.063–6.397)	0.698
Charlson co‐morbidity index			
None (0)	97	—	
Mild/moderate (1–4)	12	2.418 (0.507–11.526)	0.268
Severe (≥ 5)	15	5.898 (0.499–69.714)	0.159
Cannabis status			
Never used	15	—	
Current user	88	0.265 (0.043–1.647)	0.154
Ex‐user	21	0.542 (0.079–3.704)	0.532
CBMP route of administration			
Oil/sublingual formulations only	21	—	
Vapourised flower only	64	1.745 (0.312–9.773)	0.526
Both	39	1.428 (0.234–8.707)	0.700
Total CBD dosage			
≤ median dose (26.25 mg/day)	60	—	
> median dose (26.25 mg/day)	64	1.665 (0.579–4.791)	0.345
Total THC dosage			
≤ median dose (192.25 mg/day)	60	—	
> median dose (192.25 mg/day)	64	0.988 (0.294–3.314)	0.984
