# Correction to “UK Medical Cannabis Registry: A Clinical Outcomes Analysis for Autism Spectrum Disorder”

**DOI:** 10.1002/npr2.70172

**Published:** 2026-09-08

A. Aggarwal , S. Erridge , M. Varadpande , et al., “UK Medical Cannabis Registry: A Clinical Outcomes Analysis for Autism Spectrum Disorder,” Neuropsychopharmacology Reports 46, no. 3 (2026): e70146, 10.1002/npr2.70146.42387975 PMC13323844

Due to a production error, the individual author disclosures provided in the accepted manuscript were inadvertently replaced by the standard statement, “The authors declare no conflicts of interest.”

This section currently reads as follows:

“The authors declare no conflicts of interest.”

The section should instead have included individual disclosures for each author, as follows:

“Arushika Aggarwal and Madhur Varadpande are medical students at Imperial College London. Simon Erridge is a resident doctor, Research Director at Curaleaf Clinic, and Research Fellow at Imperial College London. Evonne Clarke is Patient Care Director at Curaleaf Clinic. Katy McLachlan is Chief Pharmacist at Curaleaf Clinic. Ross Coomber is a consultant orthopaedic surgeon at St George's Hospital, London, and Operations Director at Curaleaf Clinic. Muhammed Asghar, Urmila Bhoskar, Matthieu Crews, Andrea De Angelis, Muhammad Imran, Fariha Kamal, Laura Korb, Gracia Mwimba, Simmi Sachdeva‐Mohan, Gabriel Shaya, and James Rucker are consultant psychiatrists at Curaleaf Clinic. Mikael Sodergren is a consultant hepatopancreatobiliary surgeon at Imperial College NHS Trust, London, a Clinical Associate Professor at Imperial College London, and Chief Medical Officer of Curaleaf International. None of the authors holds shareholdings in pharmaceutical companies.”

The authors and the journal thank Kenjiro Shiraishi for bringing this production error to the journal's attention.

